# Biotechnological potentials of halophilic microorganisms and their impact on mankind

**DOI:** 10.1186/s43088-022-00252-w

**Published:** 2022-05-31

**Authors:** Bhramar Dutta, Rajib Bandopadhyay

**Affiliations:** Department of Botany, The University of Burdwan, Purba Bardhaman, West Bengal 713104 India

**Keywords:** Bioactive compounds, Biotechnological applications, Halophilic microorganisms, Osmoadaptation

## Abstract

**Background:**

Halophiles are extremophilic organisms represented by archaea, bacteria and eukaryotes that thrive in hypersaline environment. They apply different osmoadaptation strategies to survive in hostile conditions. Habitat diversity of halophilic microorganisms in hypersaline system provides information pertaining the evolution of life on Earth.

**Main body:**

The microbiome-gut-brain axis interaction contributes greatly to the neurodegenerative diseases. Gut resident halophilic bacteria are used as alternative medication for chronic brain diseases. Halophiles can be used in pharmaceuticals, drug delivery, agriculture, saline waste water treatment, biodegradable plastic production, metal recovery, biofuel energy generation, concrete crack repair and other sectors. Furthermore, versatile biomolecules, mainly enzymes characterized by broad range of pH and thermostability, are suitable candidate for industrial purposes. Reflectance pattern of halophilic archaeal pigment rhodopsin is considered as potential biosignature for Earth-like planets.

**Short conclusions:**

This review represents important osmoadaptation strategies acquired by halophilic archaea and bacteria and their potential biotechnological applications to resolve present day challenges.

**Graphical Abstract:**

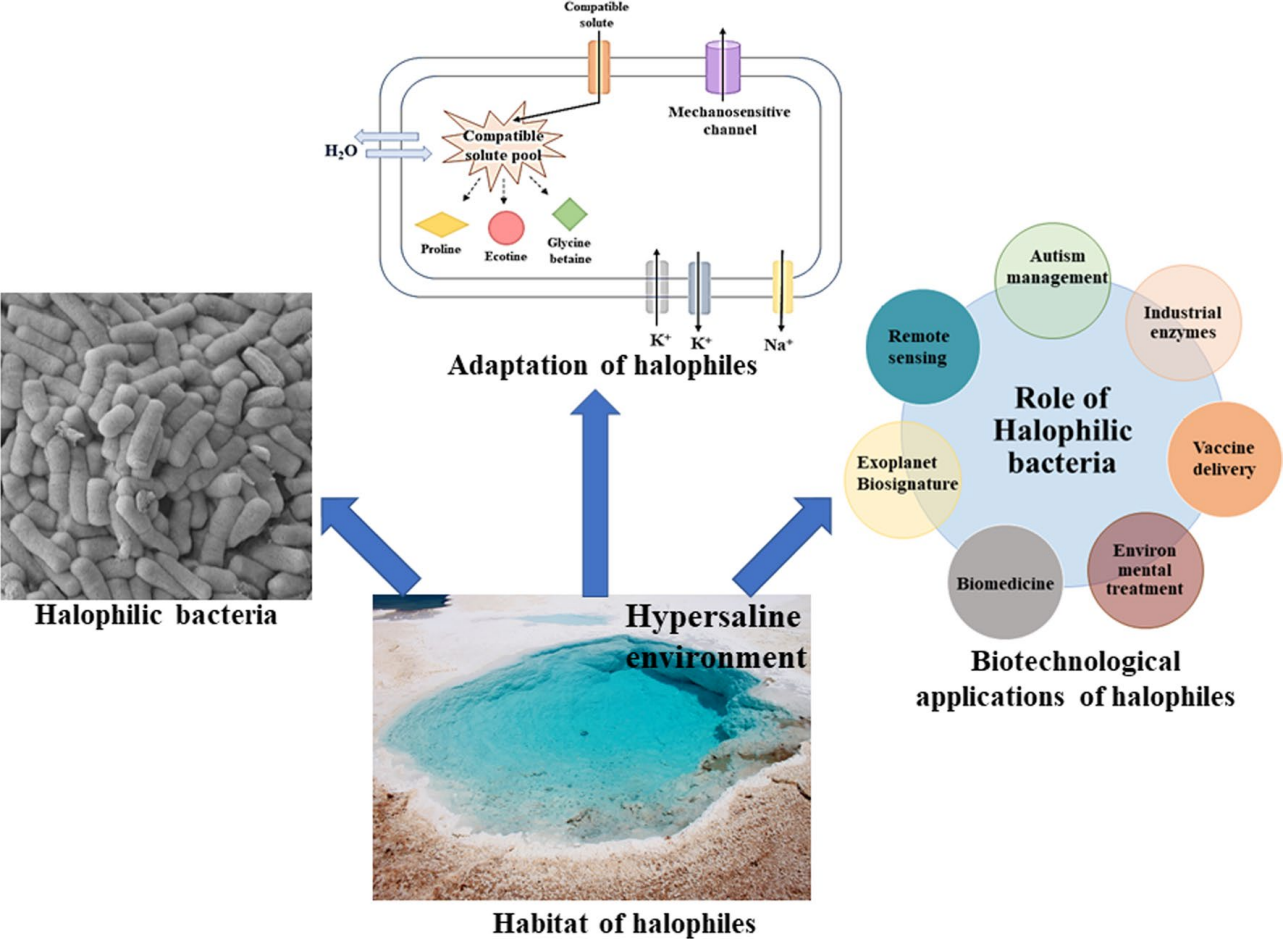

## Background

Microorganisms inhabiting different types of extreme environmental niches have been termed as thermophiles, psychrophiles, piezophiles, xerophilies, acidophile, alkaliphiles and halophiles. This successful occupancy of life form in such adverse environment have drawn the attention to the scientific community for potential applications of their bioactive molecules to be used in medicine, agriculture, bioenergy and other industries. Among them, halophilic microorganisms are characterized by the presence of major life forms in saline environment. Halophiles require a minimum of 1 M salt for growth and can proliferate in diverse range of salt concentrations. Depending on optimal salt requirement for growth they are classified as- slight (0.34–0.85 M NaCl), moderate (0.85–3.4 M NaCl) and extreme halophiles (3.4–5.1 M NaCl) [1]. A halotolerant microorganism has no absolute requirement of salt, is able to grow in the presence or absence of high salt concentration. Extreme halotolerant microorganisms are considered to grow above 2.5 M salt.

Halophiles that are mainly thrive under broad spectrum of extreme environments like-pH, salinity, temperature  are considered as poly-extremophilic microorganisms. Haloalkaliphilic bacteria such as *Desulfonatronospira thiodismutans*, *Deltaproteobacteria* sp. are adapted to grow under high salt and alkaline pH [2].

Some extreme halophiles are able to grow at high temperature solar salterns like *Methanopyrus kandler*, *Halorhabdus utahensis*. [3]. *Methanopyrus* sp. grows optimally at 110 °C at 3 M NaCl. Psychrotolerant halophile, *Chromohalobacter* *salexigens* grows 4 M NaCl at 0 °–35  °C  and produce 5-hydroxyectoine to avoid desiccation [4]

Haloalkalothermophiles elucidate some unique biochemical features of their bioactive molecules which offer high catalytic activity, remarkable stability of the compounds [3].

With the help of advanced metabolic engineering tools, efficient microbial cell factory has been developed. Expression of the ice-nucleation gene (inaZ) from *Pseudomonas syringae* established in *Halomonas* strains resulting recombinant proteins that have great potential as alternative cell factories [5].

Study of halophilic and halotolerant prokaryotes (archaea and bacteria) is often neglected and seems to be less promising than eukaryotic alga *Dunaliella salina* or fungus *Wallemia ichthyophaga*. The halophilic properties are widespread in all three domains of life *i.e*., bacteria, archaea, and eukarya. Although cellular life in hypersaline system is represented largely by prokaryotes, eukaryotic organisms are also common. Halophiles can easily be isolated from subterranean solar brine, Rajasthan, India [6]; salt marshes, saline soil saltern crystallizer ponds in Eilat, Israel; hypersaline soda lake, Magadi in Kenya, Africa; brine pool in the Sinai Peninsula, Egypt [7]; microbial mat in a pond of Mediterranean coast; Salin-de-Giraud in France [8] and Great Salt Lake or Dead Sea in Utah, USA. In a diverge scenario, cold saline lakes in the Vestfold Hills region of ice-free areas in east Antarctica have contributed resourceful insights of hypersaline life [9].

Osmoregulatory mechanism of halophilic microbes to proliferate constantly in acute salt concentration by stabilizing osmolytes or by ion balance with external environment, brought halophiles into a new arena of biotechnological applications. Considering the fact of desiccation tolerance, and survival for long span of time, it is now thoroughly studied in the discipline of astrobiology, assuming their similar existence in Mars. Adaptation for ionic cytoplasm of specially modified acidic ribosomes and stabilizing protein machinery are making them exciting choice for industrial enzyme processing. Bioactive substances are of countless pharmacological interest and therapeutic values. Halophilic lactic acid bacteria (LAB), *Tetragenococcus halophilus* has been reported from soy sauce moromi in Japan. This LAB possesses an immunomodulatory activity that exerts T-helper type -1 (Th1) immunity in humans. Thus, development of probiotics with this strain seems favourable for controlling allergic rhinitis in human [10]. Glycolipids compounds exhibiting antiviral activity against enveloped viruses, isolated from halotolerant *Rhodococcus* sp., can be a potential candidate in the treatment of herpes simplex virus (HSV-1) and human corona viruses (SARS-CoV-2) [11]. Along with this, haloarchaea, *Natrialba* sp. produce C50 carotenoid bacterioruberin, which showed robust activity against hepatitis C virus (HCV) and hepatitis B virus (HBV) [12]. This unique property could be used to formulate drugs for cancer and viral hepatitis.

This review employed survival strategies of halophilic bacteria and archaea to withstand extreme salt stresses and the biomolecules produced are of great interest for biotechnological purposes. Overall, the review work generated evidences to discover basic life principles hidden under salt both in this planet earth and beyond where they can live reasonably.

## Main text

### Long-term adaptation to saline environments

Hypersaline environment has remained a reservoir of life since the primordial days of life on earth. In those early days, salinity was one of the first chemical stresses encountered by early life and therefore, halophiles have evolved some strategies to maintain protoplasmic viability and ion balance. The mechanism used by salt-tolerant microorganisms to withstand the high saline environment are diverse as well. Some general phenomena are as follows-

**Fig. 1:**
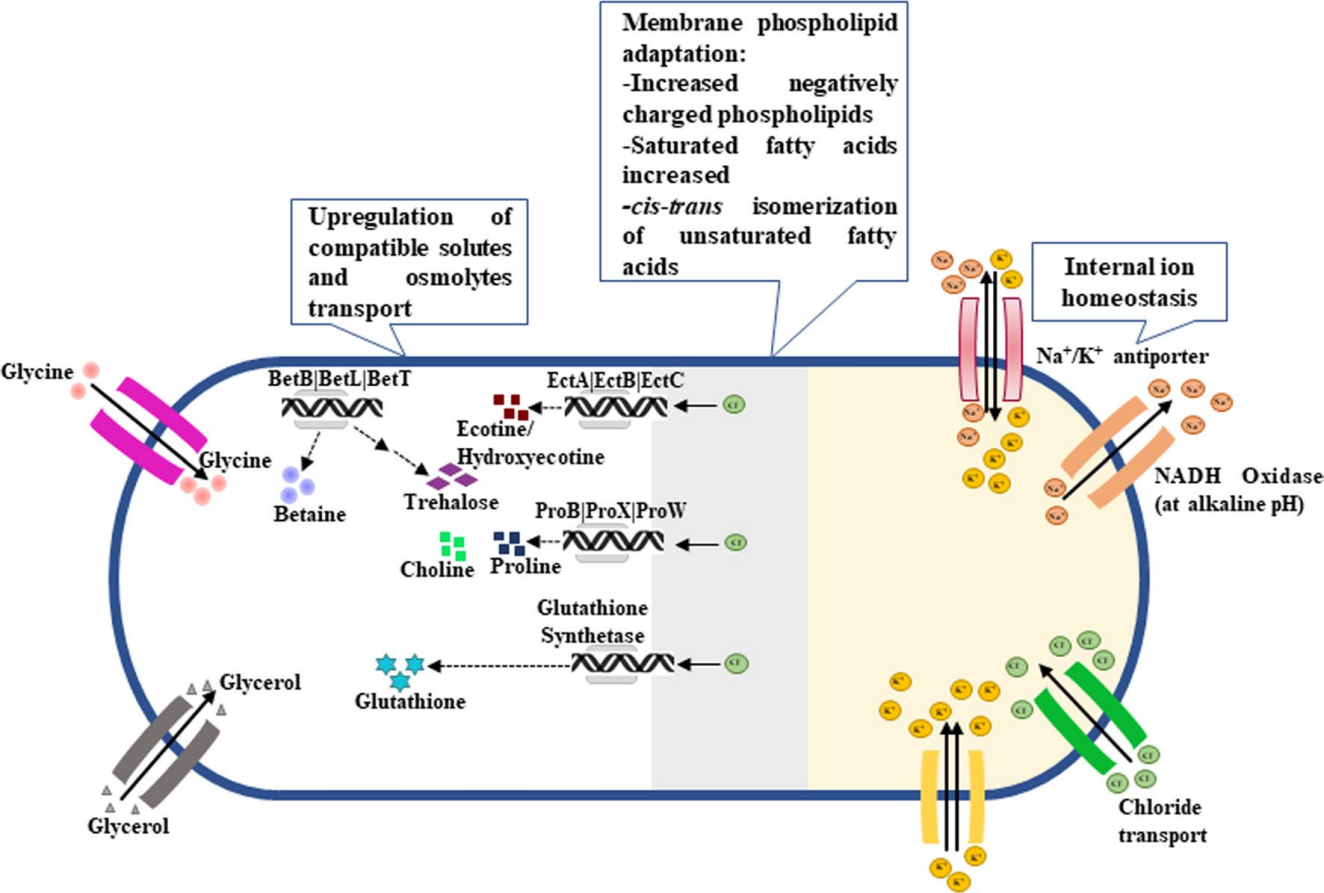
Schematic representation of the cellular processes involved in salt adaptation in halotolerant microorganisms

(i) Halophilic bacteria like, *Halobacillus halophilus*, have adapted mainly two mechanisms underlying salt stress- “salt in/out cytoplasm” to maintain osmotic balance and accumulation of compatible solutes or “organic-osmolytes” such as glycine betaine, ectoine and hydroxyectoine, which are depicted in Fig. 1. Hypo-osmotic shock protection by aquaporins and cryoprotectants has also been served along with these two primary mechanisms.
(ii) Intracellular sodium and potassium ion concentrations are regulated by light driven inward directed chloride pump halorhodopsin in *Halobacterium salinarium* and *Natronomona spharaonic* [13]
(iii) The halotolerant unicellular green algae *Dunaliella salina* can tolerate high salinities (0.5–5 M NaCl) by forming large amount of organic solute, glycerol in a salt-dependent manner [14]. *Dunaliella* sp. maintain low salt concentrations in their cytoplasm as well as in their chloroplast. Glycerol increases osmotic pressure of the cytoplasm by increasing the accumulation of other cytoplasmic solutes, thus preventing fluid loss [14].
(iv) Reduction in hydrophobic amino acids frequencies and greater propensity to form random coiled structure over *α* helices, is undoubtably determining factor of saline adaptation [15].
(v) Halophilic fungi, *Wallemia ichthyophaga* maintain low intracellular Na^+^ concentrations by accumulating glycerol as main compatible solute. Smaller amounts of arabitol and mannitol were also reported [16].

### Biotechnological importance of halophilic archaea and bacteria for mankind

This study appraises the application of halophilic microbes emphasizing on archaeal and bacterial role.

#### Potential management for neurodegeneration

Autism Spectrum Disease (ASD) encompasses symptoms like impairment in language, deficit in socialization and hyperactive repetitive stereotypic behaviour. Autism was first defined by Leo Kanner in 1943 as a neurodevelopmental disorder among children [17]. According to United States Centres for Disease Control and Prevention (CDC), approximately 1 in 59 children are diagnosed with ASD [18]. Excessive oxidative stress and lower antioxidant capacity are the two causes of this disease. ASD become one of the most challenging disorders due to lack of successful curative practices till date.

**Fig. 2:**
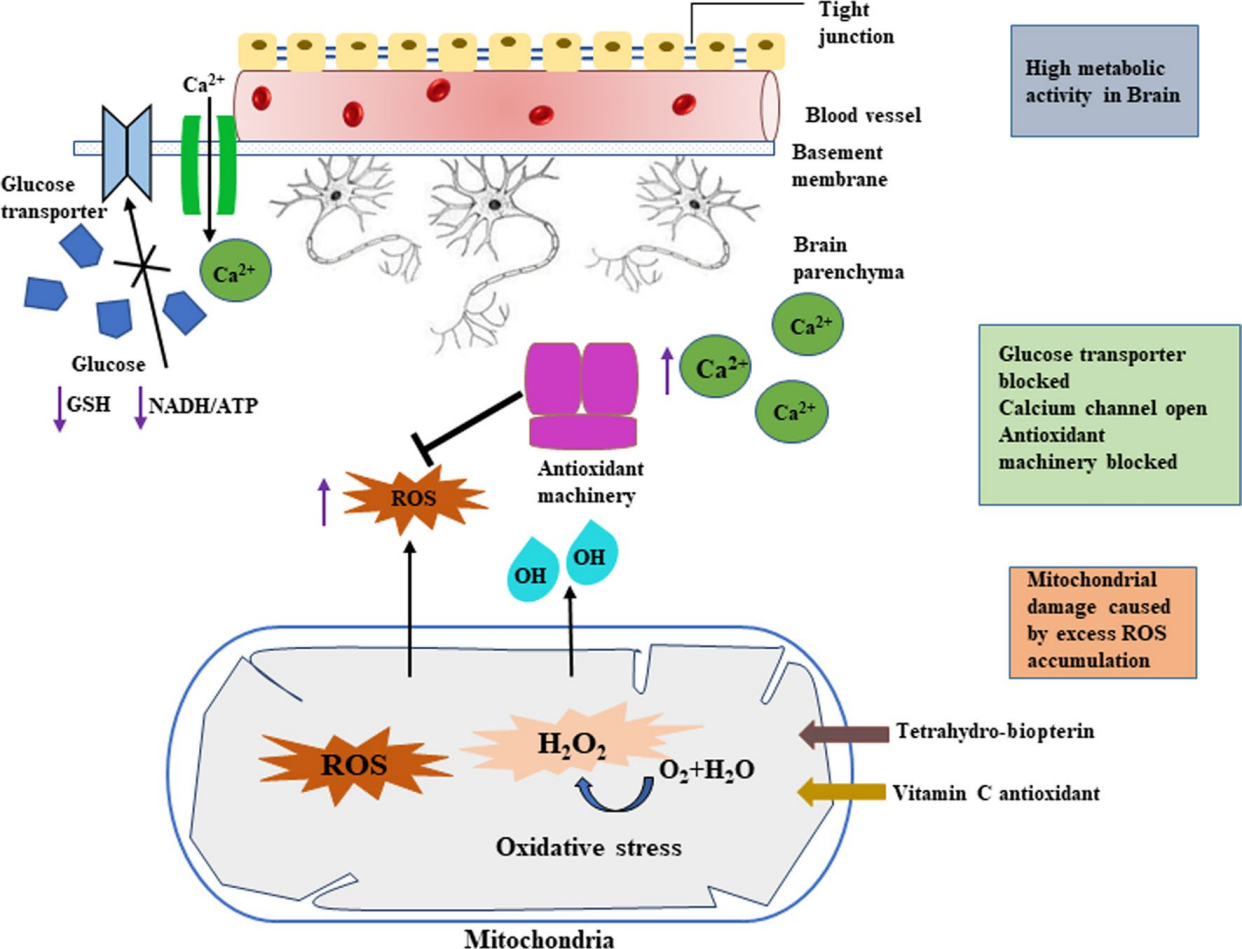
Schematic diagram showing interaction of Mitochondrial ROS and Brain activity in Autism Spectrum Disorder (ASD) patients

The disequilibrium state between prooxidant species and antioxidant defence causes mitochondrial dysfunction. The interconnected pathways of folate, methionine and glutathione metabolism severely impaired which leads to oxidative stress. A dramatic surge occurs in the levels of Reactive oxygen species (ROS) and Reactive nitrogen species (RNS). Brain is the most sensitive organ for oxidative damage caused by ROS because of its constant requirement of oxygen for its function, low antioxidants defences and high content of omega-3 polyunsaturated fatty acids (PUFAs) attributed to be oxidized. Figure 2 depicts the interaction of ROS and brain activity in ASD patients.

The alteration of gut microflora composition with enriched halophilic bacteria has increasing possibilities as a therapeutic approach of disease control [19]. Recent study emphasizes that brain activity is directly linked with gut microbiota [20]. Halophiles maintain metabolic homeostasis by accumulating compatible solutes including quaternary amines (glycine, betaine, ectoine), polyols (arabitol, glycerol, sorbitol) and sugar derivatives (trehalose, sucrose, glucosylglycerol) that manifests immune, enteric, and neural pathways. Thus, multidirectional crosstalk system between gut-brain axis (GBA) modulates the level of oxidative stress by increasing prooxidant components or reduced antioxidant enzymes capacity [21]. Another mechanism involved is the production of short chain fatty acids (SCFAs) by gut colonizing bacteria like *Enterococcus*, *Klebsiella* and *Staphylococcus*. SCFAs are mostly acetate (C_2_), propionate (C_3_), and butyrate (C_4_) compounds produced through carbohydrate fermentation which enters the central nervous system (CNS) by crossing blood–brain barriers and modulate ASD by altering the level of neurotransmitters [22].

The Human Microbiome Project Consortium identified predominantly Firmicutes and Bacteroidetes phyla in gut [23]. There are novel techniques which abandon brain disorders by modulating gut microflora. The discovery of editing technique by clustered regularly interspaced short palindromic repeats (CRISPR) and the associated nuclease 9 has a wide array of possibilities to manipulate the human microbiome [24]. Faecal microbiota transplantation (FMT) therapy with halophilic microorganism could be a promising treatment for autism [25].

#### Pigment for remote sensing

Halophilic microorganisms produce various types of pigments which protect their delicate cells from external damages. Carotenoid in *Deinococcus* sp. serve as protective agent against oxidative damage. Red pigment in *Rubrobacter radiotolerans* itself acts as an effective antioxidant. Dynamic spatial–temporal variations of pigments can be measured through satellite remote sensing system. Specific algorithms are used to process multispectral raw data monitored from satellite sensors. Halophilic archaea, *Halobacterium salinarium*, produce red colouration due to presence of β-carotene, bacterioruberins (C50 analogues of carotenoids), lycopene and diphytanyl-glycerol pigments. *Haloferax volcanii* possess bacterioruberin which confers pink hues in Dead Sea, Jordan. Bacterial genera, *Salinibacter*, thrive in hypersaline lakes and solar salterns with non-photosynthetic retinal pigments, salinixanthin and xanthorhodopsin [26]. Atmospheric circulation pattern of The Great Salt Lake, USA can be estimated by the reflectivity changes of red-coloured patches from the International Space station.

**Fig. 3:**
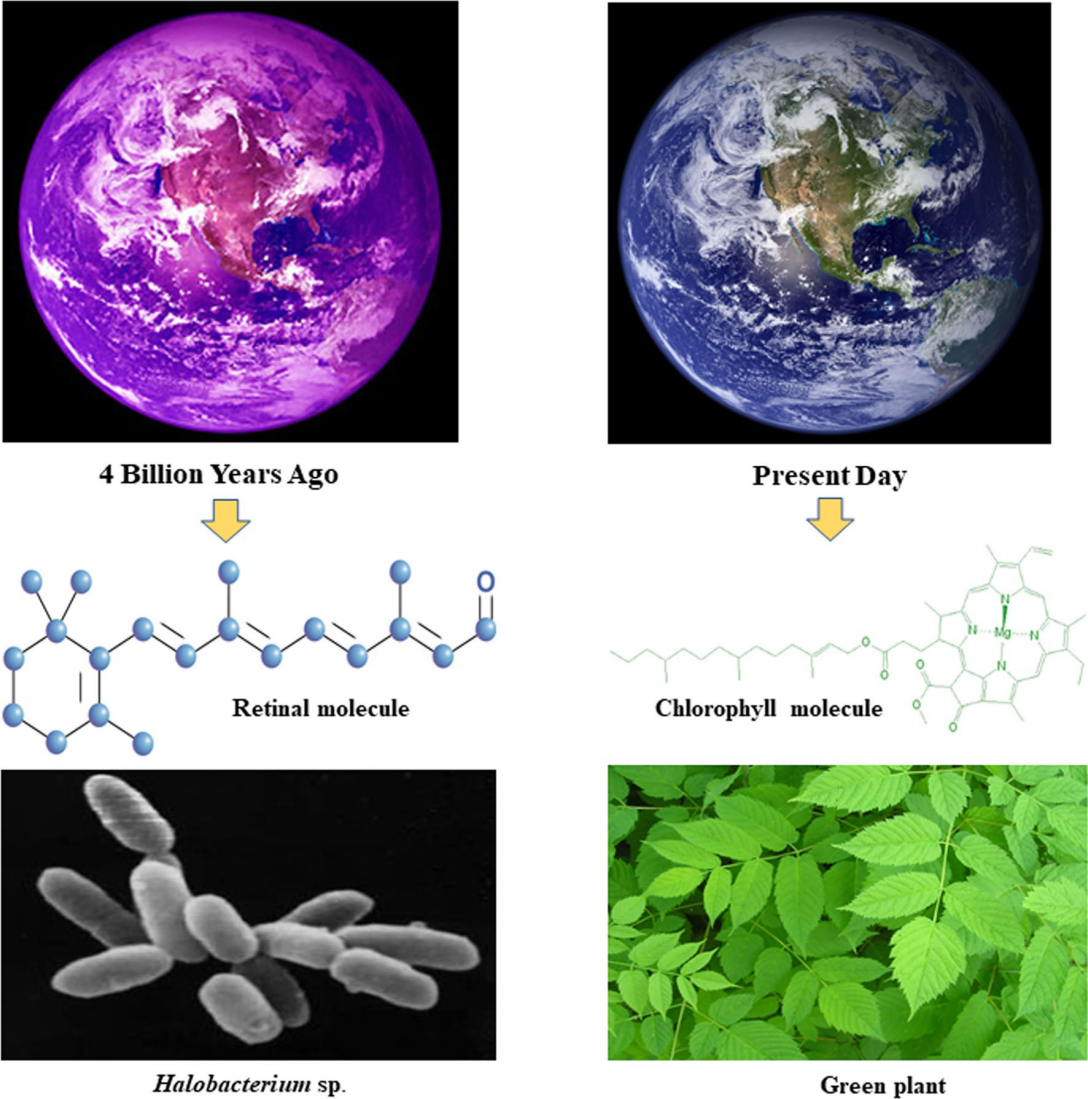
Evolution of purple earth to today’s green vegetation [the composite picture was made by using following sources: *Halobacetrium* NRC-1-https://en.wikipedia.org/wiki/Halobacterium; Purple earth-DasSarma and Schwieterman (2019); Satellite Images of the Whole Earth**-**NOAA’s Geostationary Operational Environmental Satellites release image]

Haloarchaeal retinal (C20) is a bacterioruberins purple coloured pigment, similar to higher plant photosynthetic pigments, a valuable reflectance database from visible to near-infrared region of spectrum produced by chromophore. Dense population of bacterial bloom on ocean surface can be observed by satellite, which reveals thermohaline circulation current [27]. These surface reflected radiation and time-dependent modulations are detectable biosignatures. Purple tinged molecule supposed to be dominant during the Archean eon (~ 3.0 Gyr ago) in the life form of purple bacteria [28]. “Purple Earth Hypothesis” was conceived by DasSarma, which said that halophilic bacteria used this purple-tinged retinal molecule to harness energy from the sun [29]. Chlorophyll, the main photosynthetic pigments of plants absorb red and blue wavelength of light and reflects green ones. Chlorophyll molecule may have been preceded into earth from this retinal governed life form in earth (Fig. 3). Today, retinal is found in the plum-coloured membrane of photosynthetic bacteria which absorb green light and give off the purple colour. Polyextremophilic microorganisms suggest this similar model would be potential biosignature to trace possible biological history away from our planet Earth.

#### Production of biofuel

Current energy system needs a huge revolution to meet the twenty-first century’s demands which must be economically efficient and environment friendly. The fossil fuels are running out at an alarming rate, the depletion is forcing to search for renewable bioenergy as alternative power sources. Widespread implementation of first generation (1G) biofuels is limited due to its high costs and barely developed technology. The second generation (2G) of biofuel based on cellulosic energy is enhancing energy security. Different forms of biofuels are bioethanol, biobutanol, biogas, hydrogen, and biodiesel. Bioethanol is the most suitable substitute among all the biofuels. Halophiles directly involved in ethanol and butanol production by sugar fermentation technique in hypersaline areas around the globe. *Nesterenkonia* sp. is one of its kind, reported to produce butanol and acetone under aerobic and anerobic fermentation [30]. Fendrich noticed that small amount of ethanol and acetate are produced from gram-positive, obligate halophilic bacterium, *Clostridium halophilum* in strictly anerobic condition [31]. By undergoing the anaerobic digestion process, halophiles produce large quantities of biogas. Lipase and cellulase from haloarchaeal strain *Haloarcula*, are being used in biodiesel production [32], it is estimated that 80% of biodiesel production can be achieved successfully from the archaea. Supplementation with ectoine, derived from haloarchaea promote cell growth and 57.1% increase in volumetric ethanol production [33]. Halotolerant bacterial consortia of *Joostella marina*, *Algoriphagus ratkowskyi* and *Halomonas meridiana* can degrade plant based lignocellulosic material which in turn converted into bioethanol by hydrolysis and fermentation [34].

#### Enzymes from halophilic microorganisms and their industrial applications

The adaptation of protein stability under osmotic pressure, high pH and salinity attracts many industries as best suited candidate for enzymes. Halophiles produce stable enzymes that bring success to all industrial biotechnology goals. Table 1 summarizes some useful enzymes produced by microorganisms under salinity and their potential uses in biocatalyst industries. Numerous hydrolase enzymes have scope to make third generation (3G) biofuel from algal biomass.

**Table 1:** A summarized overview of some enzymes produced by halophilic microorganisms and their industrial importance

Enzyme	Source organism	Conditions for maximum activity	Maximum activity	Uses	References
Protease	*Halococcus agarilyticus*	20% NaCl, 0.2% tween 80	49.5 U mL^−1^	Leather processing, food, dairy	[35]
	*Halobacterium* sp.				[35]
	*Haloferax mediteranei*				[35]
	*Natrial bamagadii*				[35]
	*Natronomonas pharaonic*				[35]
	*Pseudoalteromonas* sp.	55 °C, pH 8.5, 1 M NaCl	60% of relative activity		[36]
Amylase	*Haloarcula* sp.	pH 7.0, temp 45 °C, 3 M NaCl, 0.2 M MgCl_2_	Specific activity of 2975 U/mg	Food, textile, brewing industry	[37]
	*Bacillus* sp.	pH 8.0, temp 40 °C, K_m_ value 5.1 mg/mL and V_max_ value 116.28 μM/min/mL	130.53 ± 2.0 U/mL		[38]
Lipase	*Haloferax mediterranei*	pH 7.0 and 60 °C , K_m_ and V_max_ for HML toward olive oil were 1.01 mM and 1195 U/mg, respectively	50 U/mL	Detergent, textile, pharmaceuticals	[39]
	*Marinobacter* sp.	2.5–3.5 M NaCl, pH 8.5, temperature 55 °C	49.5 U mL^−1^		[40]
	*Chromohalobacter canadensis*	4 M NaCl, 50 °C and pH 7	60.57 U/mg		[41]
	*Streptomyces* sp.	pH at 10.5	7.5 U/mL		[42]
Esterase	*Hararcula marismortui*	N/D	N/D	Synthesis of optical compound, perfume, antioxidant	[43]
	*Halomonas gudaonensis*	pH 9 and temperature 30 °C, Kcat value of 12.30 s^−1^	63% of relative activity		
	*Salinicoccus roseus*	pH 4.0, 3.5 g/L of MgSO4, and tributyrin concentration of 1(%v/v)	N/D	Decolourization of synthetic dyes	[44]
Urease	*Haloarcula* sp.	N/D	N/D	Alcoholic beverages, haemodialysis	[45]
	*Salicola* sp.				
	*Idiomarina* sp.	N/D	0.9 mmol min^−1^		[46]
	*Halomonas meridiana*				
Pullalanase	*Salinivibrio* sp.	N/D	N/D	Saccharification process	[47]
	*Bacillus* sp.				[47]
	*Halorubrum* sp.	pH 7.0 and 40°C, Km 4mg/mL	7.5U/mL		[48]
Xylanase	*Halorubrum saccharovorum*	37 °C, pH 5, of 20% NaCl	4.791 U/mg of protein	Pulp and paper industry	[49]
	*Gracilibacillus* sp.	3.5% NaCl, pH 7.5 and temperature of 60 °C	541 U/g dry weight of substrate		[50]
	*Flammeovirga pacifica*	1.5 mol/L of NaCl 30 °C, pH 7.5	2.14 U/mg of specific activity		[51]
Chitinase	*Halobacterium salinarum*	1.5 M NaCl, 40 °C at pH 7.3	0.49 ± 0.005 U/mL	Waste management, food industry	[52]
DNase	*Bacillus stratosphericus*	N/D	N/D	Protein purification	[53]
	*Pseudomonas halophila*				
	*Thalassobacillus devorans*				

Where N/D indicates not determined

#### Metal bioremediation by nanoparticles

Remediating metal contaminants and waste by biogenic nanoparticles (NPs) from haloarchaea and halobacteria is a potential solution for metal pollution [54]. Halophilic bacteria are resistance to metals due to enzymatic detoxification, extra and intracellular precipitation and energy-dependent efflux system [55]. Table 2 summarizes various nanoparticles synthesized by halophiles.

**Table 2:** Details of nanoparticles synthesized by various halophiles

Source organism	Cell/by products used for nanoparticle synthesis	Nano particle types	Morphology	Size (nm)	Mechanism	Applications	References
*Halococcus salifodinae*	Whole cell	Tellurium nanoparticles (TeNPs)	Needle shaped	Diameter of 10 nm and length of 44 nm	Tellurite reductase was responsible for tellurite resistance and nanoparticle synthesis	Antibacterial activity against gram-negative and gram-positive bacteria	[54]
*Bacillus megaterium*	Whole cell	Selenium nanoparticle	Spherical	200 nm	N/D	Reduction in selenite	[56]
*Halomonas eurihalina*	Whole cell	Graphene oxide (GO)	Doubled layered graphene sheet	≈ 2.7 nm	Bacterial anaerobic reduction of GO	Enzyme encapsulation, biomaker of cancer	[57]
*Halobacillus* sp.	Whole cell	Cadmium quantum Dots (Cd-QDs)	Regular polyhedral	3.56 nm	Interaction of sulphide (S2 −) and metal ion	Biomedical applications	[58]
*Salinicoccus* sp.	Whole cell	Lead and nickel nanoparticle	N/D	Lead 80–100, nickel 10–20 nm	N/D	Heavy metal bioremediation	[55]
*Halobacterium* sp.	Gas vesicles	Gas vesicle nanoparticles (GVNPs)	Spindle-shaped vesicles	N/D	Gene fusion	Bioengineering for vaccine development	[59]
*Halomonas maura*	Exopolysaccharide	ZnS:Mn-Quatum dots	Nanoclusters	10 − 20 nm	Anionic binding	Fluorescent agent for in vitro imaging	[60]
*Geomicrobium *sp.	Extracellular enzymes (protease)	Zinc oxide nanoparticles (ZnONPs)	N/D	70 nm	Ionic interaction	Increasing stability of protein	[61]
*Halomonas salifodiane*	Polyhydroxyalkanoates PHA	Poly (3 HV-co-3HB)-based nanoparticles	Polymeric granules	179 ± 12.1 nm	Emulsification–diffusion mode of PHA and nanoparticles	Antibacterial agent	[62]
*Cupriavidus necator*	Polyhydroxybutyrate (PHB)	Silver nanoparticles (AgNPs)	Spherical	76–95 nm	Nucleation effect of nanoparticles	Biodegradable plastics, antimicrobial agent against the food-borne pathogens	[63]

Where N/D indicates not determined

Numerous essential factors like pH, temperature, salt concentration, size of NPs can affect the properties of NPs. Multitude of these factors influence the efficacy of NPs [64]. Reaction media pH played important role in morphology, stability and growth of nanoparticles. It was noted by Mishra et al. that reduction in selenite stopped above pH 8.0 in *Bacillus* sp. [56]. Metal reduction efficiency attainted maximum at pH.

Concentration of salt (% of NaCl) has a specific effect on the process of NP synthesis from halophilic microorganisms. Selenium nanoparticles synthesized by *Bacillus megaterium* affected by NaCl concentration in the solution. Decrease in selenite reduction activity above 7% NaCl [56]. Similar observation was noted in *Halobacillus* sp. showing resistance to CdCl_2_ (1.375 mM) at 22% NaCl [58]. This could be due to the negatively charged residues in salt bridges of halophilic protein may involve a multipoint adherence of positively charged residues on the nanoparticle surface which contribute greater stability of the NPs [61].

Nanofabrication process required ambient temperature for successful NP conjugation. Increased temperature above 32  °C in graphene oxide led marginal effect in reduction to graphene [57].

The quality and biological applications of NPs depend on particle size. The colour change during AgNPs agglomeration associated with the size of AgNPs in *Cupriavidus necator* [63]. Raveendran et al. have noticed a shift in the UV absorption peak for ZnS:Mn-Quatum dots due increment of the NP size above 5 nm [60].

#### Small peptides as potential biomedicine

Antimicrobial peptides (AMPs) are 12–100 amino acids long, *α*-helical, positively charged, amphiphilic molecules. Halocins are type of antimicrobial peptides, produced naturally by extreme halophilic archaea and released out into the environment. Bacteria produces ribosomally synthesized small peptide molecules (less than 100 amino acid residues), bacteriocin which can inhibit the growth of other bacteria [65]. Bacteriocins exhibit wide range of antimicrobial activity against several antibiotic resistant planktonic bacteria and has broad inhibition spectrum against yeasts, insects and mammals (Table 3).

**Table 3:** Different anti-microbial peptides (AMPs) produced by halophiles

Short peptides	Source organism	Mechanism	Applications (in biomedicine)	References
Cyclic dipeptides (CDPs)	*Pseudomonas aeruginosa*	Cyclodipeptides (CDPs) are capable of inducing apoptosis	Inhibitory effect towards plant pathogens, and human pathogen	[66]
Halocin H4	*Haloferax mediterranei*	Production of exopolysaccharides (EPS)	Inducer or activator of DNA uptake	[65]
Microcin E492	*Klebsiella pneumoniae*	Induce apoptosis in human cell lines	Bactericidal activity to Enterobacteriaceae	[67]
Streptomonomicin (STM)	*Streptomonospora alba*	Mutation of response regulator gene (*walR)*	Against *Bacillus anthracis*	[68]
Nisin	*Lactococcus lactis*	Positive charged molecule that causes pore development in the cell membrane of the target organism and thereby results in cytoplasmic membrane depolarization	Anticancer agent	[69]

The rise of multi drug resistant (MDR) bacterial strains necessitates the search for new platforms for treatment of human diseases. Bacteriocins nowadays are considered as alternative medicine against these microbial pathogens. Class I bacteriocins, also called lantibiotics, possesses post translationally modified unusual amino acid lanthionine. Halophilic bacteria, Staphylococcus simulans produce Nukacin, a variant of bacteriocin, which shows bacteriostatic action to *Bacillus subtilis* [70]. A two-peptide bacteriocin produced by vancomycin resistant strains of Enterococcus faecalis is active against foodborne pathogen *Listeria* spp. [71]. Halobacteria produces halocin during transition stage from exponential to stationary phase of growth. H6/H7 type of halocin from *Haloferax gibbonsii* can inhibit Na + /H + antiporter in mammalian cell. Halocin H6 decreases infarct size during myocardial ischemia and protect reperfusion injury of heart [72]. This finding call renaissance of medical science in terms of organ transplantation. Both H4 and C8 types of halocins from *Halobacterium* can alter the shape of rod cells into spherical cells in sensitive strains, which leads to cell lysis [73]. Numerous virus-host interactions beyond domain boundaries in hypersaline environment are possible because of halocin. The bacteriocin compound are being used to control infectious bacteria [74].

Subtilisin-A from *Bacillus firmus* exhibits anti-microbial (against pathogenic bacteria like *Staphylococcus aureus*, *Corynebacterium* *diphtheria* and *Pseudomonas aeruginosa*), antifungal (*Aspergillus niger*, *Aspergillus flavus*), antioxidant activities (against DPPH and nitric oxide radicals) and anti-cervical cancer activity (against HeLa cell line with IC50 at 53 μg/mL). Thus, bioactive AMPs could be used in therapeutics [75].

#### Formulation of new generation of liposomes

Liposomes are microscopic vesicular structures consisting of lipid bilayers with an internal fluid compartment which can encapsulate and deliver small molecule drugs or vaccines to a specific target site in the body. Archaeosomes are liposomal formulation made up with ether linked total polar lipids (TPL) of the domain Archaea, conferring high stability of liposomes [76]. Sulfated glycolipid gas vesicles from *Halobacterium salinarum* elicit both antibody and cell mediated immunity. Isoprenoid glycerolipid vesicles from *Methanobrevibacter smithii* stimulate the activation of APC mediated expression of both MHC class I and II, induce co-stimulatory molecules and make cytotoxic responses to CD8 + cell. Both macrophage and dendritic cell evoke a memory generation even in the absence of CD4 + helper T lymphocytes [77]. Higa et al. developed ultradeformable liposome from *Halorubrum tebenquichense* which can enhance skin permeation ability and could be carrier of transdermal delivery for procyanidins [78]. The rapid, low-cost drug delivery approach represents haloarchaea an excellent candidate for treating allergies, cancer and neoplastic diseases as nano-delivery based vaccination. But a major limitation of liposome mediated drug delivery lies in quality assurance and cost. Sometime phospholipid of liposome hydrolyzed by protease. Future research will overcome the existing challenges and surpass the current limitations.

#### Repairment of concrete crack caused by road salt

**Fig. 4:**
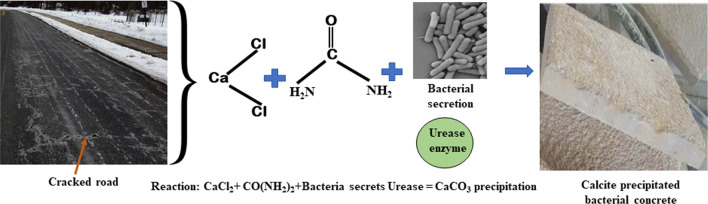
Halophilic bacteria used in road crack repairing

Snow and ice removal is one of the most crucial things in winter to keep moving road traffic in the coldest countries in the world. Anti-icing brine solution containing sodium chloride, magnesium chloride and calcium chloride are applied to combat icing period. The application of de-icing road salt began in the 1930s and it became widespread in highway maintenance during the 1960s [79]. It has been reported that 20 million tons of highway salts were being used in the year 2016 in USA only [80]. NaCl is used mostly as anti-freeze because it is the least expensive and easily available and works at temperatures above − 12 °C. Road salt run-off has created severe ecological changes and affected the soil microflora in many countries. Salt-laden water interact with root systems and produce adverse effects in local vegetation. Halophilic bacteria are able to survive in high pH (pH 9.0) and grows in negative osmotic potential. Few studies have been reported the impact of excess salt in microbial communities in terms of soil fertility. Archaeal genera, *Natrinema* and *Haloterrigena* are known for soil stabilization [81]*.* Figure 4 shows halophilic bacteria *Sporosarcina pasteurii* plays fundamental role in biomineralization by converting calcium ions from dumped road soil by urease enzyme into calcite or calcium carbonate also known as limestone. This bacterium aided in biocementing the cracks in concrete by depositing CaCO_3_ precipitation [82]*.* Halophilic bacteria, *Pseudomonas putida* and *Vibrio fischeri* (standard bacterial test) have been used to estimate the toxicity level and the physical and chemical properties of soil [83]. As a permanent member of the microbiota in de-icing salts, halophilic bacteria plays a central role in soil ecosystems.

#### Waste management by biodegradation of hydrocarbon and plastics

Halophiles are involved in biodegradation of wide range of hydrocarbons. Biological treatment of saline wastewater by heterotrophic, halotolerant microbes catches tremendous demand in industrial scale. Basic evolutionary study suggests oldest halophilic microbial diversity found in stromatolite during Precambrian period resemble modern microbial mat growing in hypersaline lakes [84]. *Marinobacter* is a metabolically versatile hydrocarbon degrading taxon. Halotolerant bacteria, *Alcanivorax*, *Burkholderia*, *Pandoraea* and *Enterobacter* can degrade broad spectrum of petroleum hydrocarbons like benzene, ethylbenzene, *n*-alkanes (C6–C40) compounds and xylene into carbon dioxide [85]. Salt tolerant bacteria, *Pseudomonas putida* and *Ralstonia pickettii* predominantly chemotactically swim towards toluene and mineralize benzene and phenol. Archaeal strain, *Haloferax*, *Halobacterium*, and *Halococcus* from hypersaline Gulf area use benzoic acid salicylate as sole carbon and energy source and converted to catechol under aerobic condition. Biotransformation of hydrocarbon produce several higher-value intermediate products like benzyl alcohol, phenolic compounds and catechol. Potent natural antioxidant hydroxytyrosol (HTyr) produced by *Halomonas* sp. strain HTB 24, is isolated from hypersaline olive mill wastewater. *Pseudomonas aeruginosa*, isolated from crude oil contaminated soil can transform polyphenolic compound 4-tyrosol to HTyr, which has well documented anti-inflammatory activity [86].

Another most formidable environmental challenge today is plastic waste. The non-degradability of plastics and petrochemical-derived polymers are aggravated by extensive global use and their inaccurate management [87]. Microplastics (< 5 mm in size) are ubiquitous from deepest ocean floor to highest mountains, very recently in human blood [88].

Polyhydroxyalkanoates (PHA) are natural linear polyester that are produced by microbial fermentation processes, having similar thermochemical properties like molecular weight, semicrystallinity, piezoelectricity, low permeability for water and oxygen, melting temperature (*T*_m_) with the additional advantage of being completely biodegradable [89]. PHA synthesized by several haloarchaea and halophilic bacteria when there is availability of excess carbon sources (substrate) and limited oxygen, nitrogen supply [90]. Among the different classes of PHA’s like poly-3-hydroxybutyrate (PHB), poly(3-hydroxybutyrate-co-3-hydroxyvalerate) (PHBV), polyhydroxyvalerate (PHV), PHBs are only 100% biodegradable ones, thus offer the best solution to protect environment from plastic pollution [89]. During intracellular degradation, 3PHB is oxidized by dehydrogenase and converted into acetyl-CoA by *β*-ketothiolase to form non -toxic substances.

A number of Gram positive and Gram negative non-halophile PHA producing bacteria have been reported. Among them, *Pseudomonas putida*, *Burkholderia thailandensis*, *Alcaligenes latus*, *Escherichia coli* are gaining interest due to high yield of PHA and substrate spectrum [91]. But PHA production from halophilic microorganisms have added benefits due to its low -cost purification process and minimal environmental hazards. After fermentation, downstream processing of PHA plays important role in production cost. Intracellularly produced PHA granules from halophiles can be done by osmotic lysis that burst the particles and separated easily from non-PHA cell mass (NPCM) [91]. Halophiles can utilize cheap and renewable carbon sources for PHA production. A brief summary of some important PHA producing halophiles is given in Table 4.

**Table 4:** Polyhydroxyalkanoates (PHA) production by halophiles using various substrates

Polymer	Carbon source	Microorganism	Productivity	References
Polycaprolactone	Oil palm trunk sap	*Psychrobacter* sp.	1.2 ± 0.3 g/l	[92]
PHBV and PHBV4HB	Volatile fatty acids (olive oil waste)	*Haloferax mediterranei*	1.57 ± 0.05 g/l	[93]
PHB and PHBV	Sucrose	*Halomonas elongata*	0.95 g/ l	[94]
PHA	Starch	*Haloarcula* sp.	0.1066 ± 0.3355 g/l	[95]
PHB	Glycerol	*Haloquadratum walsbyi*	N/D	[96]
PHA	Glucose	*Natronobacterium* sp.	0.1 g/l	[90]
PHA	Glucose	*Halorubrum chaoviator*	N/D	[90]
PHA	CO_2_	*Halomonas boliviensis*	1.48 g/l	[97]
PHA	Fructose	*Vibrio proteolyticus*	1.72 g/l	[98]
PHBV	Glucose	*Halococcus morrhuae*	N/D	[90]

Where N/D indicates not determined

#### Bioremediation for sustainable agriculture

Rising soil salinity is a global concern for crop production. Salinity affects all aspects of plant development in terms of morphological, physiological and biochemical processes. To combat this menace, industrial agriculture, plant growth promoting bacteria (PGPB) are used as promising alternate strategy. Bacteria belonging to different genera including *Arthrobacteria*, *Azospirilum*, *Bacillus*, *Klebsiella*, *Paenibacillus*, *Enterobacter* and *Halomonas* are commonly used halophiles which mitigate salt stress and enhance plant growth. These agronomically potential halotolerant bacteria are used as an inoculant in biocontrol of plant pathogen, production of biostimulants and making biofertilizer. PGPB improves plant growth by triggering plant growth hormones like cytokinin, accumulation of abscisic acid (ABA), degradation of ROS and enhanced nutrient uptake by root. Bacteria produce 1- aminocyclopropane-1-carboxylate (ACC) deaminase, ammonia, hydrogen cyanide (HCN), siderophore which helped the plant to reduce stress. Several bacteria have been reported to provide some degree of tolerance to host plants under salinity stress in tomato, lettuce, wheat and soybean that maintain sustainable plant health [99, 100].

### Future prospects

The search for new life elsewhere in the galaxy has gained an increasing interest over the past two decades. Astrobiologists detected most promising sign of life on the extrasolar planet, Mars, called exoplanet biosignature [101]. Data collected from ExoMars Mission (2018) confirms that Mars temporarily hosted abundant water on the surface [102]. Salty water pockets and frigid water acquirers near South Martian poles are packaged with manganese and calcium perchlorate which is most likely to be habitable for halophilic bacteria [103]. Molecular structure resembles that haloarchaeon, *Halorubrum chaoviator*, could survive in freezing and thawing brine in Mars [104]. The archaea with higher tolerance to perchlorate (upto 0.4 M), can use chlorate as terminal electron acceptor during anaerobic respiration [105]. Therefore, relative abundance of perchlorate in the Martian regolith could be analogues for understanding nitrogen fixation on extremely arid regions, like Atacama Desert on Earth. These investigations pointed that emergence and adaptation of life in salt brine during prebiotic evolution could be the earliest signature of life. Thus, halophiles could be used in future for searching extra-terrestrial life.

## Conclusions

The study of halophillicity of the microorganisms provides significant insight as the basis of potential biotechnological applications. The availability of modern techniques of bioengineering and molecular biology would enable to construct biomolecules for industrial interest. Existence of life in Earth like planets though speculative, plausible model of halophilic life has been correlated to great extent with available data, provides substantial hope for identifying new ways of research in Astrobiology. The discovery and progressive characterization of halophiles in gut-brain research have created new clinical therapies for neuro degenerative disorders. The ability of these life forms to withstand high salt concentration, osmotic pressure, pH and often a combination of these conditions, made a transforming insight to use them as a promising candidate in various industries. Moreover, production of biofuel, bioremediation of synthetic compounds and biocleaning of environment have crucial values for several green purposes in maintaining the environmental sustainability.

## Data Availability

All analysed data are available in this article.

## Abbreviations

ABA: Abscisic acid
ACC: 1- Aminocyclopropane-1-carboxylate
AMP: Antimicrobial peptides
APC: Antigen-presenting cell
ASD: Autism spectrum disease
CD + cells: Cluster of differentiation
CDC: Centres for disease control and prevention
CNS: Central nervous system
CRISPR: Clustered regularly interspaced short palindromic repeats
FMT: Faecal microbiota transplantation
GBA: Gut brain axis
HBV: Hepatitis B virus
HCV: Hepatitis C virus
HSV: Herpes simplex virus
LAB: Lactic acid bacteria
T_m_: Melting temperature
MDR: Multi drug resistant
MHC: Major histocompatibility complex
NPs: Nanoparticles
NPCM: Non-PHA cell mass
PGPB: Plant growth promoting bacteria
PHA: Polyhydroxyalkanoates
PHB: Poly-3-hydroxybutyrate
PHBV: Poly-3-hydroxybutyrate-co-3-hydroxyvalerate
PHV: Polyhydroxyvalerate
PUFA: Polyunsaturated fatty acid
RNS: Reactive nitrogen species
ROS: Reactive oxygen species
SARS-CoV-2: Severe acute respiratory syndrome coronavirus 2
SCFA: Short chain fatty acids
TPL: Total polar lipid

## References

[1] Abaramak G, Kırtel O, Öner ET (2020) Fructanogenic halophiles: A new perspective on extremophiles. In: Salwan, Sharma (eds) Physiological and Biotechnological Aspects of Extremophiles. Academic Press, pp. 123-130. 10.1016/B978-0-12-818322-9.00009-5

[2] Sorokin DY, Tourova TP, Henstra AM (2008). Sulfidogenesis under extremely haloalkaline conditions by *Desulfonatronospira thiodismutans* gen. nov., sp. nov., and *Desulfonatronospira delicata* sp. nov.–a novel lineage of Deltaproteobacteria from hypersaline soda lakes. Microbiology.

[3] Bowers KJ, Wiegel J (2011). Temperature and pH optima of extremely halophilic archaea: a mini-review. Extremophiles.

[4] Anan'ina LN, Gorbunov AA, Pyankova AA (2021). Physiological response of the moderately halophilic psychrotolerant strain *Chromohalobacter* sp. N1 to salinity change and low temperature. Can J Microbiol.

[5] de Araujo GG, Rodrigues F, Gonçalves FL, Galante D (2019). Survival and ice nucleation activity of *Pseudomonas syringae* strains exposed to simulated high-altitude atmospheric conditions. Sci Rep.

[6] Pathak AP, Cherekar MN (2015). Hydrobiology of hypersaline Sambhar Salt Lake a Ramsar site, Rajasthan, India. IJMS.

[7] Walsby AE (1980). A square bacterium. Nature.

[8] Caumette P, Matheron R, Raymond N, Relexans JC (1994). Microbial mats in the hypersaline ponds of Mediterranean salterns (Salins-de-Giraud, France). FEMS Microbiol Ecol.

[9] Bowman JP, McCammon SA, Rea SM, McMeekin TA (2000). The microbial composition of three limnologically disparate hypersaline Antarctic lakes. FEMS Microbiol Lett.

[10] Kumazawa T, Nishimura A, Asai N, Adachi T (2018). Isolation of immune-regulatory *Tetragenococcus halophilus* from miso. PLoS ONE.

[11] Esposito PF, Giugliano R, Sala GD (2021). Combining OSMAC approach and untargeted metabolomics for the identification of new glycolipids with potent antiviral activity produced by a marine *Rhodococcus*. Int J Mol Sci.

[12] Hegazy GE, Abu-Serie MM, Abo-Elela GM, Ghozlan H, Sabry SA, Soliman NA, Abdel-Fattah YR (2020). In vitro dual (anticancer and antiviral) activity of the carotenoids produced by haloalkaliphilic archaeon *Natrialba* sp. M6. Sci Rep.

[13] Yun JH, Ohki M, Park JH (2020). Pumping mechanism of NM-R3, a light-driven bacterial chloride importer in the rhodopsin family. Sci Adv.

[14] Oren A (2020). The microbiology of red brines. Adv Appl Microbiol.

[15] Elcock AH, McCammon JA (1998). Electrostatic contributions to the stability of halophilic proteins. J Mol Biol.

[16] Zajc J, Kogej T, Galinski EA (2014). Osmoadaptation strategy of the most halophilic fungus, *Wallemia ichthyophaga*, growing optimally at salinities above 15% NaCl. Appl Environ Microbiol.

[17] Kanner L (1943). Autistic disturbances of affective contact. Nervous Child.

[18] Prevalence of Autism Spectrum Disorder Among Children Aged 8 Years — Autism and Developmental Disabilities Monitoring Network, 11 Sites, United States, 2014 (2018) MMWR. CDC surveillance summaries: Morbidity and mortality weekly report. CDC surveillance summaries/Centers for Disease Control 67:1–23. 10.15585/mmwr.ss6706a1 10.15585/mmwr.ss6706a1 PMC5919599 29701730

[19] Massaoudi YO, Ciobica A, Dobrin I, El Hassouni M (2019). Halophilic bacteria as a potential management for autism. Rom Biotech Lett.

[20] Svoboda E (2020). Could the gut microbiome be linked to autism?. Nature.

[21] Dumitrescu L, Popescu-Olaru I, Cozma L (2018). Oxidative stress and the microbiota-gut-brain axis. Oxid Med Cell Longev.

[22] Mirzaei R, Bouzari B, Hosseini-Fard SR (2021). Role of microbiota-derived short-chain fatty acids in nervous system disorders. Biomed Pharmacother.

[23] Huttenhower C, Gevers D, Knight R (2012). Structure, function and diversity of the healthy human microbiome. Nature.

[24] Sander JD, Joung JK (2014). CRISPR-Cas systems for editing, regulating and targeting genomes. Nat Biotechnol.

[25] Evrensel A, Ceylan ME (2016). Fecal microbiota transplantation and its usage in neuropsychiatric disorders. Clin Psychopharmacol Neurosci.

[26] Oren A (2013). *Salinibacter*: an extremely halophilic bacterium with archaeal properties. FEMS Microbiol Lett.

[27] Grimes DJ, Ford TE, Colwell RR (2014). Viewing marine bacteria, their activity and response to environmental drivers from orbit. Microb Ecol.

[28] Sanromá E, Pallé E, Parenteau MN (2013). Characterizing the purple earth: modelling the globally integrated spectral variability of the archean earth. Astrophys J.

[29] DasSarma S, Schwieterman EW (2019). Early evolution of purple retinal pigments on earth and implications for exoplanet biosignatures. Int J Astrobiol.

[30] Amiri H, Azarbaijani R, Yeganeh LP (2016). *Nesterenkonia* sp. strain F, a halophilic bacterium producing acetone, butanol and ethanol under aerobic conditions. Sci Rep.

[31] Fendrich C, Hippe H, Gottschalk G (1990). *Clostridium halophilium* sp. nov. and C. *littorale* sp. nov., an obligate halophilic and a marine species degrading betaine in the Stickland reaction. Arch Microbiol.

[32] Li X, Yu HY (2013). Halostable cellulase with organic solvent tolerance from *Haloarcula* sp. LLSG7 and its application in bioethanol fermentation using agricultural wastes. J Ind Microbiol Biot.

[33] Zhang L, Lang Y, Wang C, Nagata S (2008). Promoting effect of compatible solute ectoine on the ethanol fermentation by *Zymomonas mobilis* CICC10232. Process Biochem.

[34] Cortes-Tolalpa L, Norder J, van Elsas JD, Salles JF (2018). Halotolerant microbial consortia able to degrade highly recalcitrant plant biomass substrate. Appl Microbiol Biot.

[35] Gaonkar SK, Furtado IJ (2018). Isolation and culturing of protease-and lipase-producing *Halococcus agarilyticus* GUGFAWS-3 from marine *Haliclona* sp. inhabiting the rocky intertidal region of Anjuna in Goa. India Ann Microbiol.

[36] Mellado E, Sánchez-Porro C, Martín S, Ventosa A (2004) Extracellular hydrolytic enzymes produced by moderately Halophilic Bacteria. In: Ventosa A (ed) Halophilic Microorganisms. Springer, Berlin, Heidelberg, pp. 285-295. 10.1007/978-3-662-07656-9_21

[37] Siroosi M, Borujeni FB, Amoozegar MA (2021). Halophilic amylase production and purification from *Haloarcula* sp. strain D61. Biointerface Res Appl Chem.

[38] Bandal JN, Tile VA, Sayyed RZ (2021). Statistical based bioprocess design for improved production of amylase from Halophilic *Bacillus* sp. H7 isolated from marine water. Molecules.

[39] Hemamalini R, Khare SK (2018). Halophilic lipase does forms catalytically active aggregates: evidence from *Marinobacter* sp. EMB5 lipase (LipEMB5). Int J Biol Macromol.

[40] Ai L, Huang Y, Wang C (2018). Purification and characterization of halophilic lipase of *Chromohalobacter* sp. from ancient salt well. J Basic Microbiol.

[41] Akmoussi-Toumi S, Khemili-Talbi S, Ferioune I, Kebbouche-Gana S (2018). Purification and characterization of an organic solvent-tolerant and detergent-stable lipase from *Haloferax mediterranei* CNCMM 50101. Int J Biol Macromol.

[42] Mohamed MA, Awad HM (2021). New lipase-producing *Streptomyces* isolated from halo-alkaline habitat in Wadi El Natrun: polyphasic identification and statistical optimization of enzyme production. Beni-Suef Univ J Basic Appl Sci.

[43] Tutuncu HE, Balci N, Tuter M, Karaguler NG (2019). Recombinant production and characterization of a novel esterase from a hypersaline lake, Acıgöl, by metagenomic approach. Extremophiles.

[44] Dutta B, Nigam VK, Panja AS, Shrivastava S, Bandopadhyay R (2021). Statistical optimisation of esterase from *Salinicoccus roseus* strain RF1H and its potential application in synthetic dye decolorisation. Biocatal Biotransfor.

[45] Zhou Y, Tang K, Wang P (2020). Identification of bacteria-derived urease in the coral gastric cavity. Sci China Earth Sci.

[46] Hosseini M, Babaha F, Al-Rubaye MT (2017). Urease-producing halophilic bacteria isolated from Bahr Al-Milh Salt Lake, Karbala, Iraq. J Pure Appl Microbiol.

[47] Elyasifar B, Arbabsolimani N, Ajudanifar H (2014). Isolation of moderately halophilic bacteria producing pullulanase enzyme from degh biarjemand desert of shahrod. Iran Iran J Public Health.

[48] Siroosi M, Amoozegar MA, Khajeh K (2014). Purification and characterization of a novel extracellular halophilic and organic solvent-tolerant amylopullulanase from the haloarchaeon, *Halorubrum* sp. strain Ha25. Extremophiles.

[49] Malik AD, Furtado IJ (2019). Cellulase-free xylanase by *Halococcus thailandensis* GUMFAS7 and *Halorubrum saccharovorum* GUMFAS1—bionts of a sponge *Cinachyrella cavernosa*. Microbiology.

[50] Giridhar PV, Chandra TS (2021). Xylanase production by halophilic bacterium *Gracilibacillus* sp TSCPVG under solid state fermentation. Res J Biotechnol.

[51] Cai ZW, Ge HH, Yi ZW (2018). Characterization of a novel psychrophilic and halophilic β-1, 3-xylanase from deep-sea bacterium, *Flammeovirga pacifica* strain WPAGA1. Int J Biol Macromol.

[52] García-Fraga B, Da Silva AF, López-Seijas J, Sieiro C (2014). Functional expression and characterization of a chitinase from the marine archaeon *Halobacterium salinarum* CECT 395 in *Escherichia coli*. Appl Microbiol Biotechnol.

[53] Moreno ML, Piubeli F, Bonfá MR (2012). Analysis and characterization of cultivable extremophilic hydrolytic bacterial community in heavy-metal-contaminated soils from the Atacama Desert and their biotechnological potentials. J Appl Microbiol.

[54] Srivastava P, Nikhil EV, Bragança JM, Kowshik M (2015). Anti-bacterial TeNPs biosynthesized by haloarcheaon *Halococcus salifodinae* BK3. Extremophiles.

[55] Diba H, Cohan RA, Salimian M (2021). Isolation and characterization of halophilic bacteria with the ability of heavy metal bioremediation and nanoparticle synthesis from Khara salt lake in Iran. Arch Microbiol.

[56] Mishra RR, Prajapati S, Das J et al (2011) Reduction of selenite to red elemental selenium by moderately halotolerant Bacillus megaterium strains isolated from Bhitarkanika mangrove soil and characterization of reduced product. Chemosphere 84:1231–1237. 10.1016/j.chemosphere.2011.05.025. 10.1016/j.chemosphere.2011.05.025 21664643

[57] Raveendran S, Chauhan N, Nakajima Y (2013). Ecofriendly route for the synthesis of highly conductive graphene using extremophiles for green electronics and bioscience. Part Part Syst Charact.

[58] Bruna N, Collao B, Tello A (2019). Synthesis of salt-stable fluorescent nanoparticles (quantum dots) by polyextremophile halophilic bacteria. Sci Rep.

[59] DasSarma S, Karan R, DasSarma P (2013). An improved genetic system for bioengineering buoyant gas vesicle nanoparticles from Haloarchaea. BMC biotechnol.

[60] Raveendran S, Girija AR, Balasubramanian S (2014). Green approach for augmenting biocompatibility to quantum dots by extremophilic polysaccharide conjugation and nontoxic bioimaging. ACS Sustain Chem Eng.

[61] Sinha R, Khare SK (2014). Differential interactions of halophilic and non-halophilic proteases with nanoparticles. Sustain Chem Process.

[62] Abd El-malek F, Rofeal M, Farag A (2021). Polyhydroxyalkanoate nanoparticles produced by marine bacteria cultivated on cost effective Mediterranean algal hydrolysate media. J Biotechnol.

[63] Castro-Mayorga JL, Freitas F, Reis MA (2018). Biosynthesis of silver nanoparticles and polyhydroxybutyrate nanocomposites of interest in antimicrobial applications. Int J Biolo Macromol.

[64] Majhi K, Let M, Kabiraj A et al (2021) Metal recovery using nanobiotechnology. In: Ghosh, Webster TJ (eds) Nanobiotechnology: microbes and plant assisted synthesis of nanoparticles, mechanisms and applications. Elsevier, pp. 283-301. 10.1016/B978-0-12-822878-4.00018-3

[65] Rodriguez-Valera F, Juez G, Kushner DJ (1982). Halocins: salt-dependent bacteriocins produced by extremely halophilic rods. Can J Microbiol.

[66] Dammak DF, Zarai Z, Najah S et al (2017) Antagonistic properties of some halophilic thermoactinomycetes isolated from superficial sediment of a solar saltern and production of cyclic antimicrobial peptides by the novel isolate *Paludifilum halophilum*. BioMed Res Intr. 10.1155/2017/1205258. 10.1155/2017/1205258 PMC5551467 28819625

[67] Hetz C, Bono MR, Barros LF, Lagos R (2002). Microcin E492, a channel-forming bacteriocin from Klebsiella pneumoniae, induces apoptosis in some human cell lines. PNAS.

[68] Metelev M, Tietz JI, Melby JO (2015). Structure, bioactivity, and resistance mechanism of streptomonomicin, an unusual lasso peptide from an understudied halophilic actinomycete. Chem Biol.

[69] Varas MA, Muñoz-Montecinos C, Kallens V (2020). Exploiting zebrafish xenografts for testing the in vivo antitumorigenic activity of microcin E492 against human colorectal cancer cells. Front Microbiol.

[70] Ceotto H, Holo H, Da Costa KF (2010). Nukacin 3299, a lantibiotic produced by *Staphylococcus simulans* 3299 identical to nukacin ISK-1. Vet Microbiol.

[71] Hu CB, Malaphan W, Zendo T (2010). Enterocin X, a novel two-peptide bacteriocin from *Enterococcus faecium* KU-B5, has an antibacterial spectrum entirely different from those of its component peptides. Appl Environ Microbiol.

[72] Lequerica JL, O’Connor JE, Such L (2006). A halocin acting on Na+/H+ exchanger of Haloarchaea as a new type of inhibitor in NHE of mammals. J Physiol Biochem.

[73] Li Y, Xiang H, Liu J, Zhou M, Tan H (2003). Purification and biological characterization of halocin C8, a novel peptide antibiotic from *Halobacterium* strain AS7092. Extremophiles.

[74] Atanasova NS, Pietilä MK, Oksanen HM (2013). Diverse antimicrobial interactions of halophilic archaea and bacteria extend over geographical distances and cross the domain barrier. Microbiologyopen.

[75] Manikandan P, Moopantakath J, Imchen M, Kumavath R, Senthil Kumar PK (2021). Identification of multi-potent protein subtilisin A from halophilic bacterium *Bacillus firmus* VE2. Microb Pathog.

[76] Sprott GD, Tolson DL, Patel GB (1997). Archaeosomes as novel antigen delivery systems. FEMS Microbiol Lett.

[77] McCluskie MJ, Deschatelets L, Krishnan L (2017). Sulfated archaeal glycolipid archaeosomes as a safe and effective vaccine adjuvant for induction of cell-mediated immunity. Hum Vacc Immunother.

[78] Higa LH, Schilrreff P, Perez AP (2012). Ultra deformable archaeosomes as new topical adjuvants. Nanomed Nanotechnol.

[79] Paschka MG, Ghosh RS, Dzombak DA (1999). Potential water-quality effects from iron cyanide anticaking agents in road salt. Water Environ Res.

[80] Bolen WP (2016) US geological survey minerals yearbook 2016: Salt. https://www.usgs.gov/centers/nmic/salt-statistics-and-information

[81] Pecher WT, Al Madadha ME, DasSarma P (2019). Effects of road salt on microbial communities: halophiles as biomarkers of road salt pollution. PLoS ONE.

[82] Gorospe CM, Han SH, Kim SG (2013). Effects of different calcium salts on calcium carbonate crystal formation by *Sporosarcina pasteurii* KCTC 3558. Biotechnol Bioproc E.

[83] Černohlávková J, Hofman J, Bartoš T et al (2008) Effects of road deicing salts on soil microorganisms. Plant Soil Environ 54:479–485. 10.17221/431-PSE

[84] Wong HL, Ahmed-Cox A, Burns BP (2016) Molecular ecology of hypersaline microbial mats: current insights and new directions. Microorganisms 4:6. 10.3390/microorganisms4010006 10.3390/microorganisms4010006 PMC5029511 27681900

[85] Margesin R, Moertelmaier C, Mair J (2013) Low-temperature biodegradation of petroleum hydrocarbons (n-alkanes, phenol, anthracene, pyrene) by four actinobacterial strains. Int Biodeter Biodegr 84:185–191. 10.1016/j.ibiod.2012.05.004

[86] Liebgott PP, Amouric A, Comte A et al (2009) Hydroxytyrosol from tyrosol using hydroxyphenylacetic acid-induced bacterial cultures and evidence of the role of 4-HPA 3-hydroxylase. Res Microbiol 160:757–766. 10.1016/j.resmic.2009.09.015 10.1016/j.resmic.2009.09.015 19837158

[87] Moharir RV, Kumar S (2019) Challenges associated with plastic waste disposal and allied microbial routes for its effective degradation: a comprehensive review. J Clean Prod 208:65–76. 10.1016/j.jclepro.2018.10.059

[88] Leslie HA, van Velzen MJ, Brandsma SH (2022) Discovery and quantification of plastic particle pollution in human blood. Environ Int. 10.1016/j.envint.2022.107199 10.1016/j.envint.2022.107199 35367073

[89] Li Z, Yang J, Loh XJ (2016). Polyhydroxyalkanoates: opening doors for a sustainable future. NPG Asia Mater.

[90] Legat A, Gruber C, Zangger K (2010). Identification of polyhydroxyalkanoates in halococcus and other haloarchaeal species. Appl Microbiol Biotechnol.

[91] Khatami K, Perez-Zabaleta M, Owusu-Agyeman I, Cetecioglu Z (2021) Waste to bioplastics: How close are we to sustainable polyhydroxyalkanoates production? Waste Manag 119:374–388. 10.1016/j.wasman.2020.10.008 10.1016/j.wasman.2020.10.008 33139190

[92] Sekiguchi T, Sato T, Enoki M (2011) Isolation and characterization of biodegradable plastic degrading bacteria from deep-sea environments. JAMSTEC Rep Res Dev 11:33–41. 10.5918/jamstecr.11.33

[93] Wang K, Zhang R (2021) Production of polyhydroxyalkanoates (PHA) by Haloferax mediterranei from food waste derived nutrients for biodegradable plastic applications. J Microbiol Biotechnol 31(2):338–347. 10.4014/jmb.2008.08057 10.4014/jmb.2008.08057 PMC9706037 33203825

[94] Cristea A, Baricz A, Leopold N (2018). Polyhydroxybutyrate production by an extremely halotolerant *Halomonas elongata* strain isolated from the hypersaline meromictic Fără Fund Lake (Transylvanian Basin, Romania). J Appl Microbiol.

[95] Karray F, Abdallah BM, Baccar N (2021). Production of poly (3-Hydroxybutyrate) by *Haloarcula*, *Halorubrum,* and *Natrinema haloarchaeal* genera using starch as a carbon source. Archaea.

[96] Bolhuis H, Martín-Cuadrado AB, Rosselli R (2017). Transcriptome analysis of *Haloquadratum walsbyi*: vanity is but the surface. BMC Genomics.

[97] Weiss TL, Young EJ, Ducat DC (2017). A synthetic, light-driven consortium of cyanobacteria and heterotrophic bacteria enables stable polyhydroxybutyrate production. Metab Eng.

[98] Hong JW, Song HS, Moon YM (2019). Polyhydroxybutyrate production in halophilic marine bacteria *Vibrio proteolyticus* isolated from the Korean peninsula. Bioprocess Biosyst Eng.

[99] Egamberdieva D, Kucharova Z (2009). Selection for root colonising bacteria stimulating wheat growth in saline soils. Biol Fertil Soils.

[100] Orhan F (2016) Alleviation of salt stress by halotolerant and halophilic plant growth-promoting bacteria in wheat (*Triticum aestivum*). Braz J Microbiol 47:621–627. 10.1016/j.bjm.2016.04.001 10.1016/j.bjm.2016.04.001 PMC4927673 27133557

[101] Marais DD, Walter MR (1999) Astrobiology: exploring the origins, evolution, and distribution of life in the universe. Annu Rev Ecol Syst 30:397–420. 10.1146/annurev.ecolsys.30.1.397 10.1146/annurev.ecolsys.30.1.397 11543275

[102] Villanueva GL, Liuzzi G, Crismani MM (2021). Water heavily fractionated as it ascends on mars as revealed by ExoMars/NOMAD. Sci Adv.

[103] Pidhorodetska D, Fauchez T, Villanueva G, Domagal-Goldman S (2020). Detectability of molecular signatures on TRAPPIST-1e through transmission spectroscopy simulated for future space-based observatories. Astrophys J Lett.

[104] Leuko S, Rettberg P, Pontifex AL, Burns BP (2014). On the response of halophilic archaea to space conditions. Life.

[105] Oren A, Bardavid RE, Mana L (2014) Perchlorate and halophilic prokaryotes: implications for possible halophilic life on Mars. Extremophiles 18:75–80. 10.1007/s00792-013-0594-9. 10.1007/s00792-013-0594-9 24150694

